# Supplementary material for: Causal inference in medical records and complementary systems pharmacology for metformin drug repurposing towards dementia
Source: Nat Commun. 2022 Dec 10;13:7652. doi: 10.1038/s41467-022-35157-w (PMC9741618; doi:10.1038/s41467-022-35157-w)
Supplement: Supplementary file 1 — Supplementary Information [file 41467_2022_35157_MOESM1_ESM.docx]

**SUPPLEMENTARY INFORMATION**

This file complements the content of the manuscript titled “Causal inference in medical records and complementary systems pharmacology for metformin drug repurposing towards dementia”. It contains 12 extended data figures and 14 extended data tables.

**SUPPLEMENTARY FIGURES**


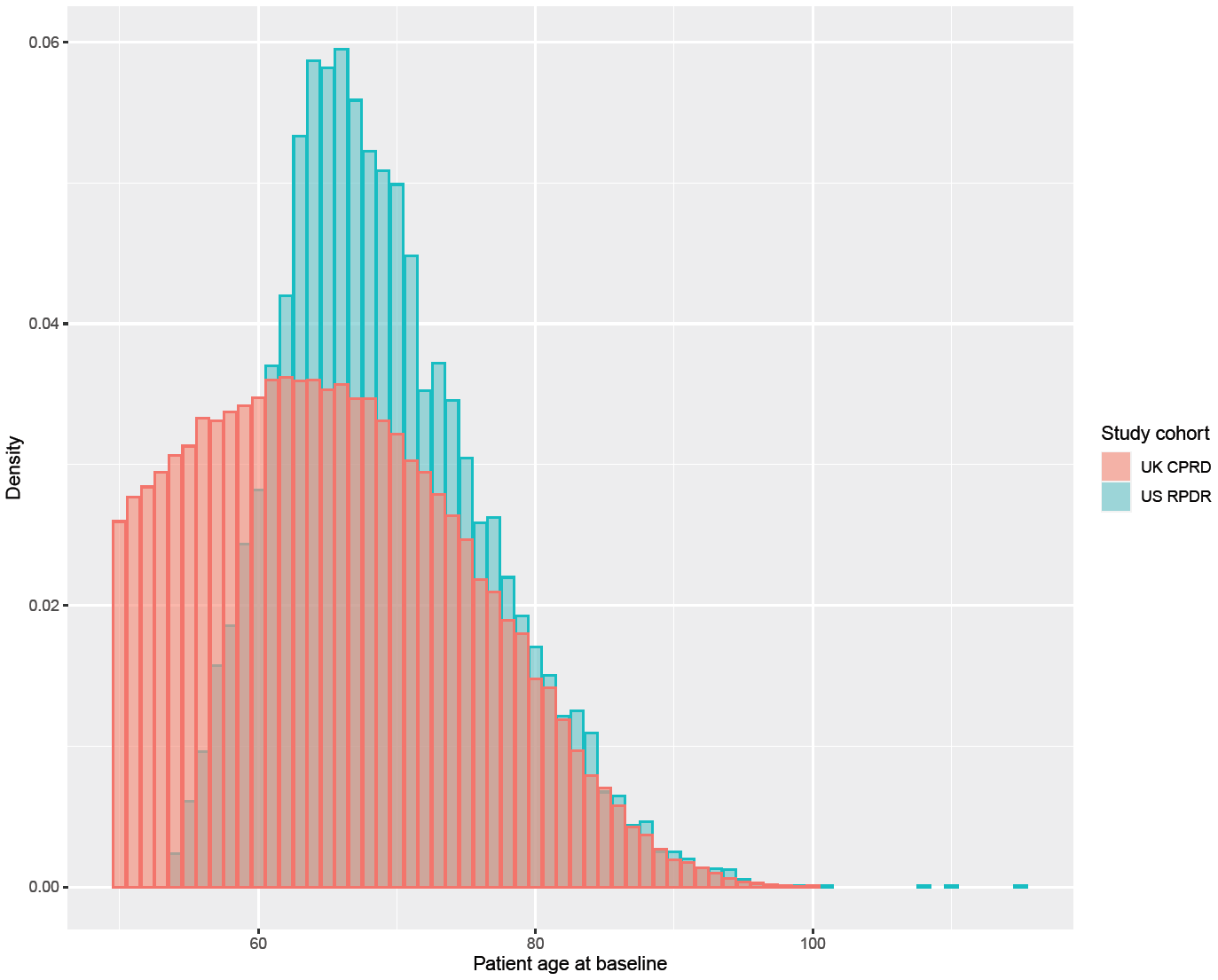


**Supplementary Figure 1 | Density curves for patient age at baseline.** Both the US RPDR (blue) and the UK CPRD cohorts (salmon) are graphed.


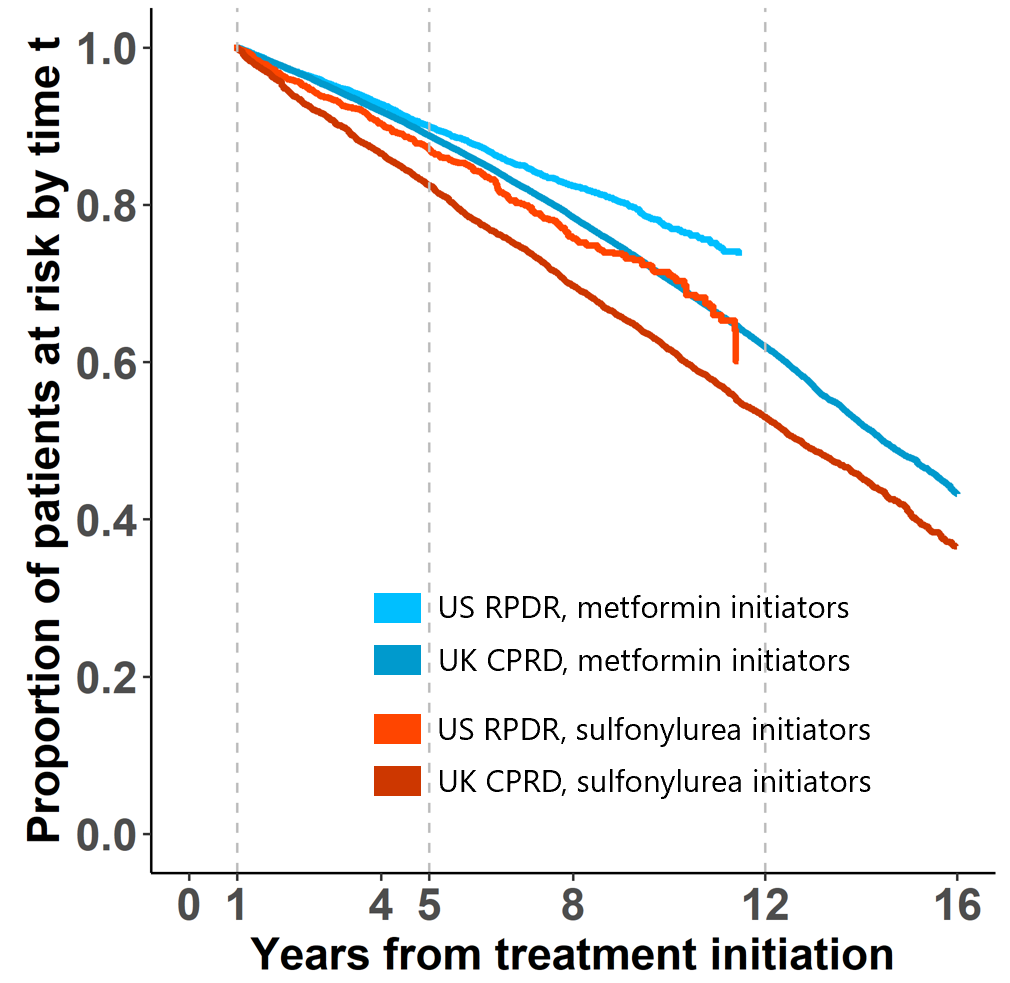


**Supplementary Figure 2 | Proportion of patients at risk over time among metformin and sulfonylurea initiators.** Both the US RPDR and the UK CPRD cohorts are graphed.


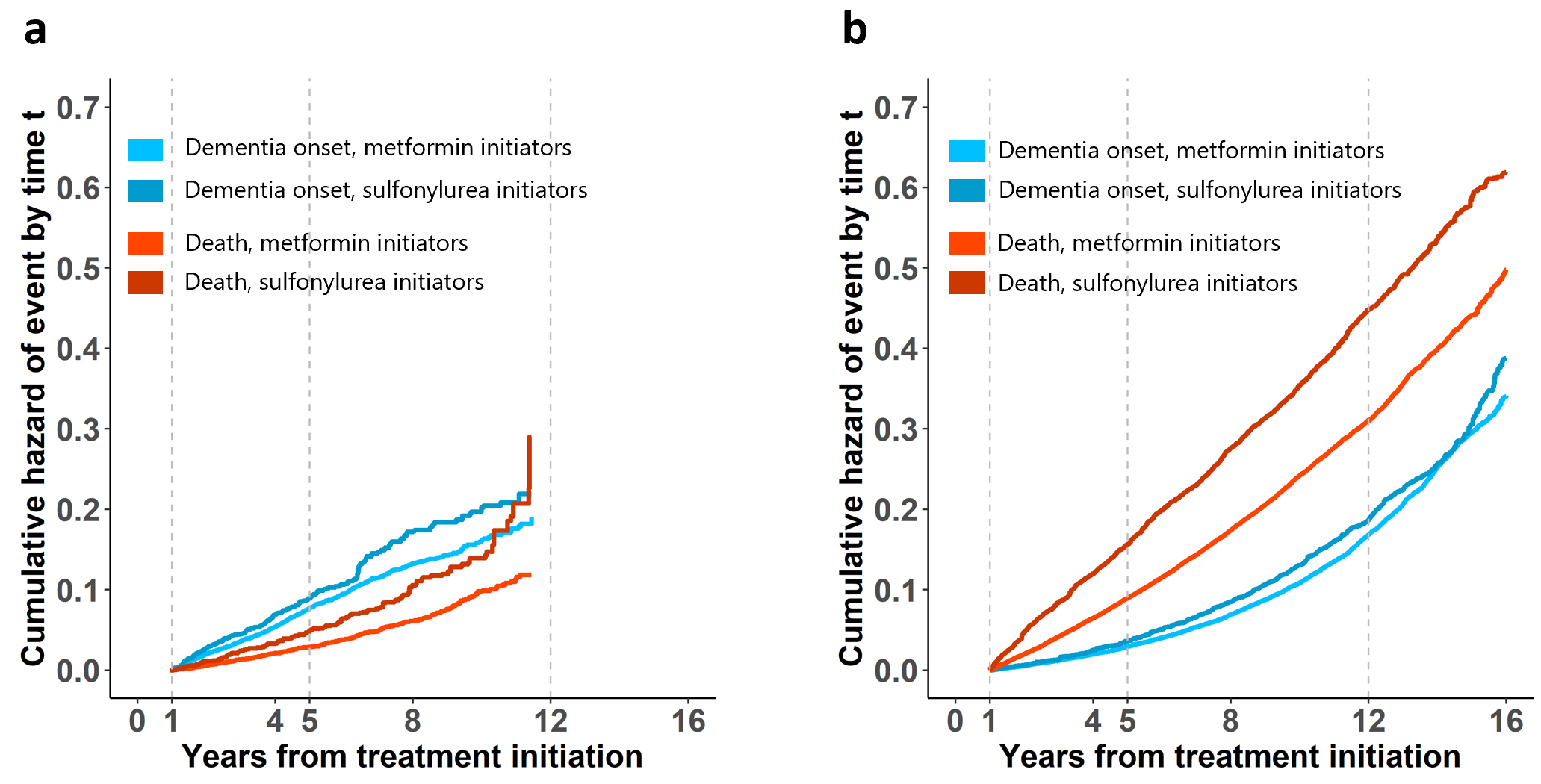


**Supplementary Figure 3 | Absolute cumulative hazard of dementia onset and death.** Cumulative risk curves in the **(a)** US RPDR and **(b)** UK CPRD cohorts for metformin and sulfonylurea initiators.


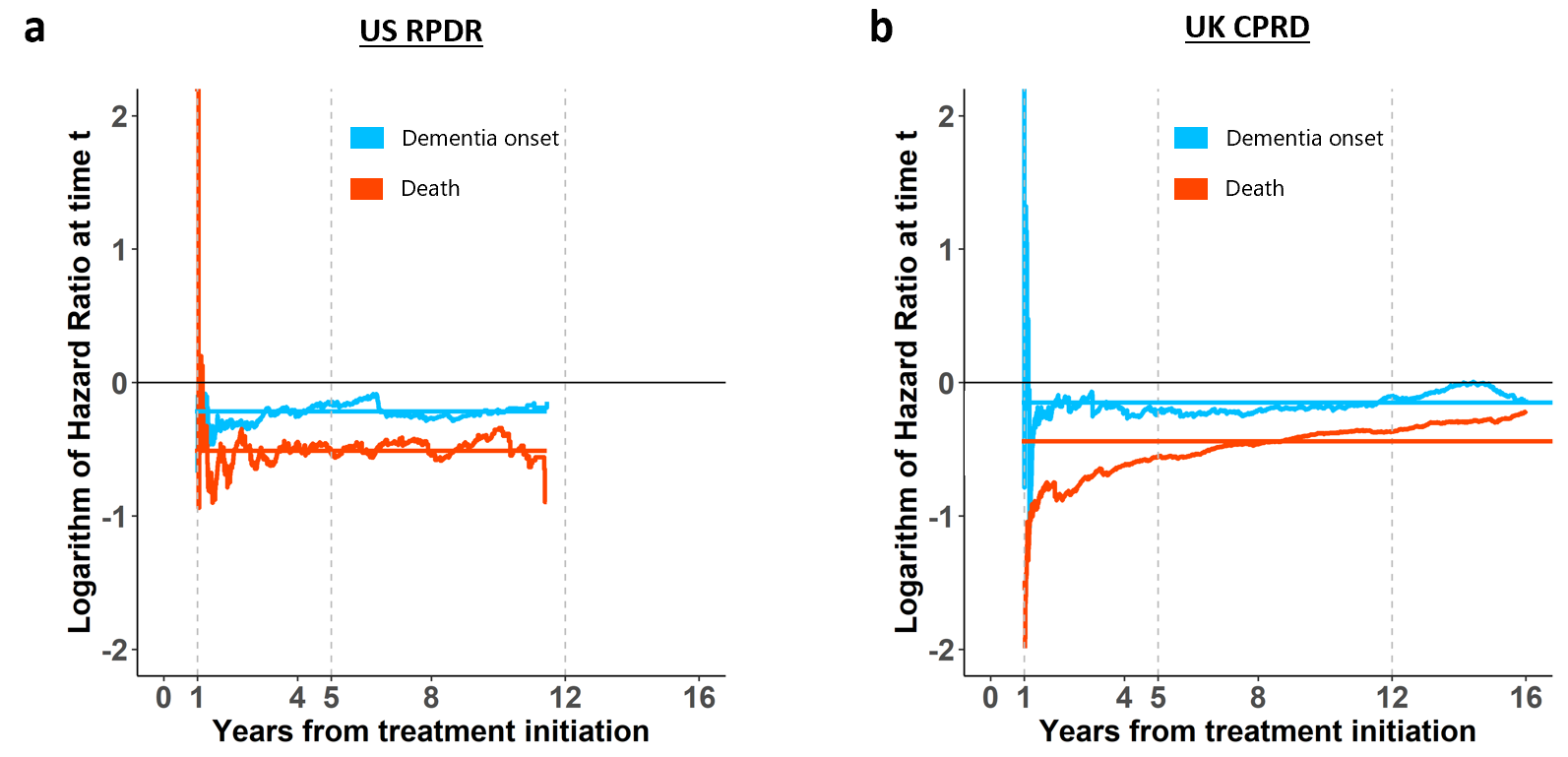


**Supplementary Figure 4 | Comparison of the log of hazard ratios estimated via the Cox PH model vs. the nonparametric approach, for both dementia onset and death. (a)** US RPDR and **(b)** UK CPRD cohorts, metformin vs. sulfonylureas (reference).


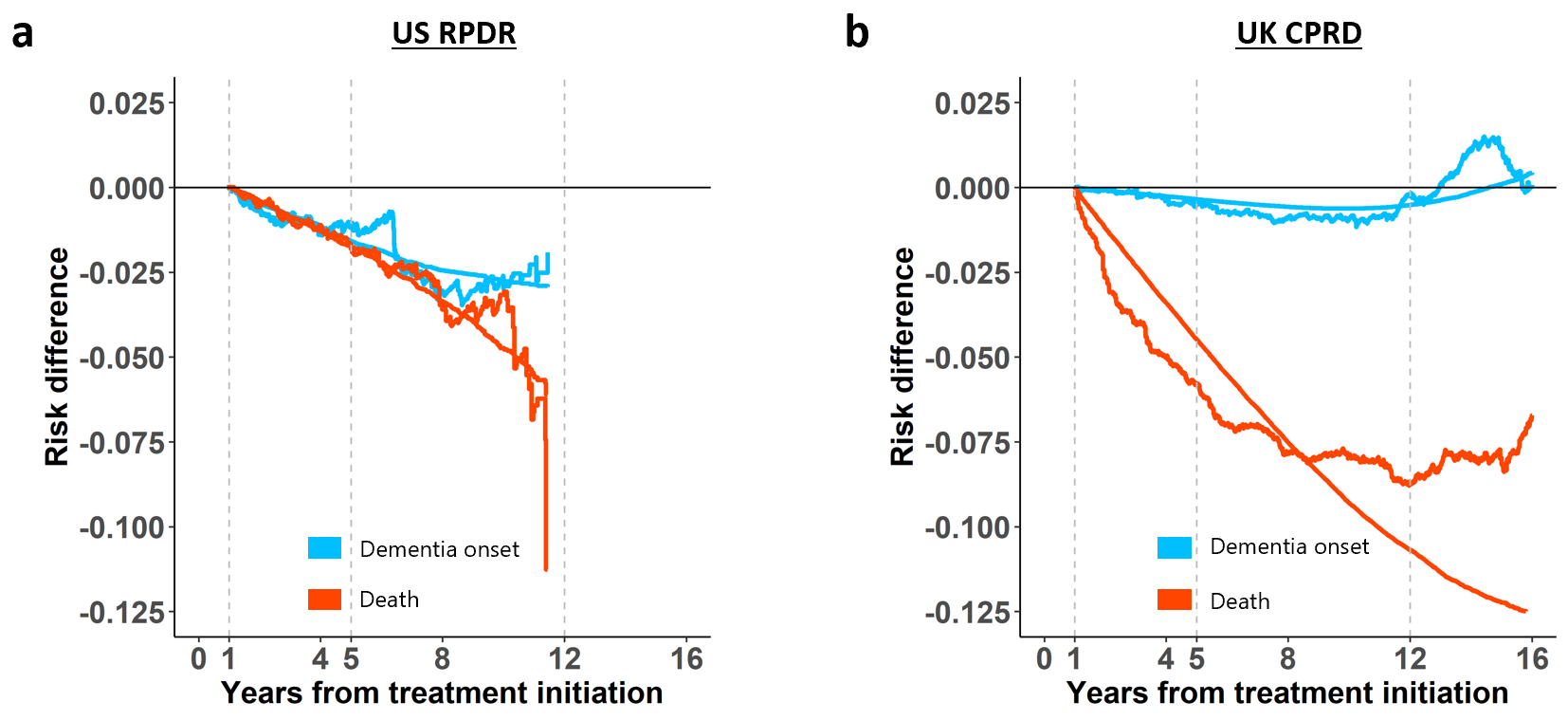


**Supplementary Figure 5 | Risk differences between metformin and sulfonylureas for two competing outcomes, dementia onset and death.** Estimates from a Cox PH model and a nonparametric approach in the **(a)** US RPDR and **(b)** UK CPRD cohorts.


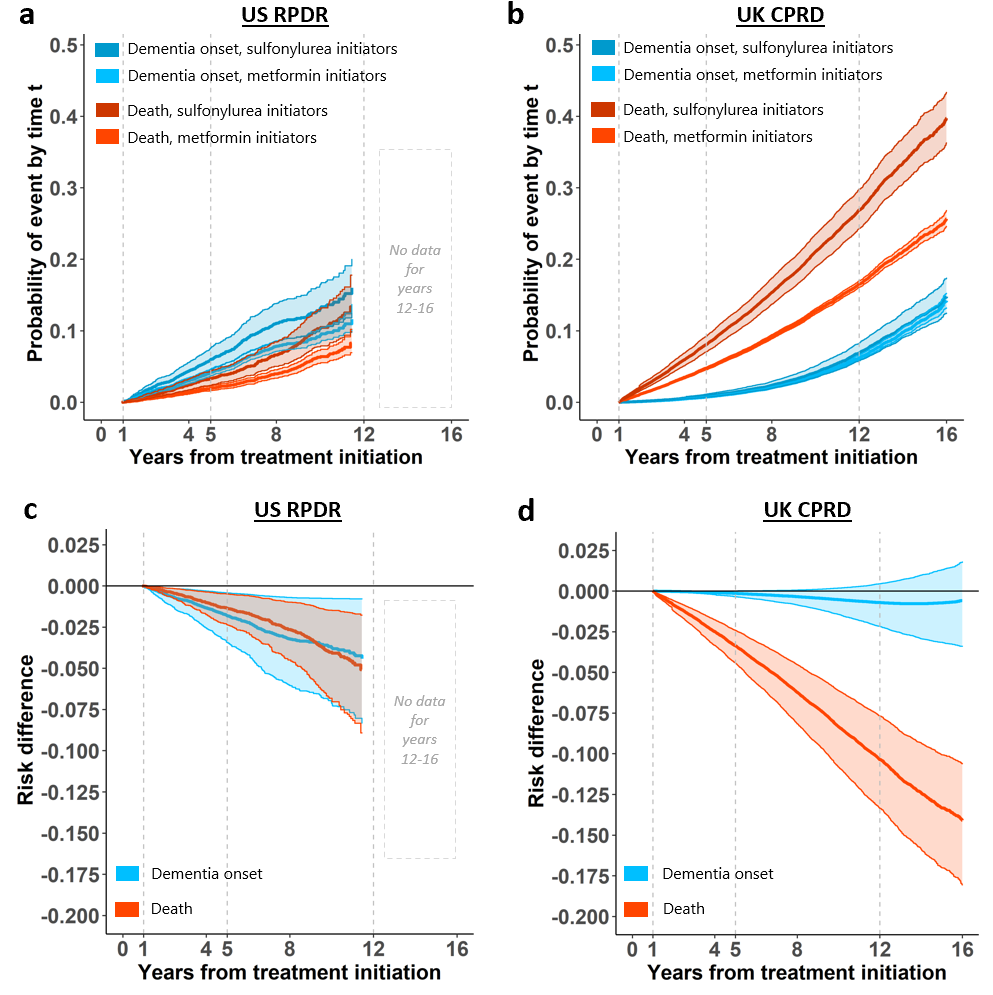


**Supplementary Figure 6 | Results of the competing risks analyses in the stratum of patients of aged ≤ 70 at baseline.** CIF curves for dementia onset and death in the **(a)** US RPDR and **(b)** UK CPRD cohorts. Risk differences between metformin and sulfonylureas (reference) in dementia onset and in death in the **(c)** US RPDR and **(d)** UK CPRD cohorts. Shaded areas represent 95% CIs, based on pointwise 0.025- and 0.975-quantiles of sample bootstrap distributions.


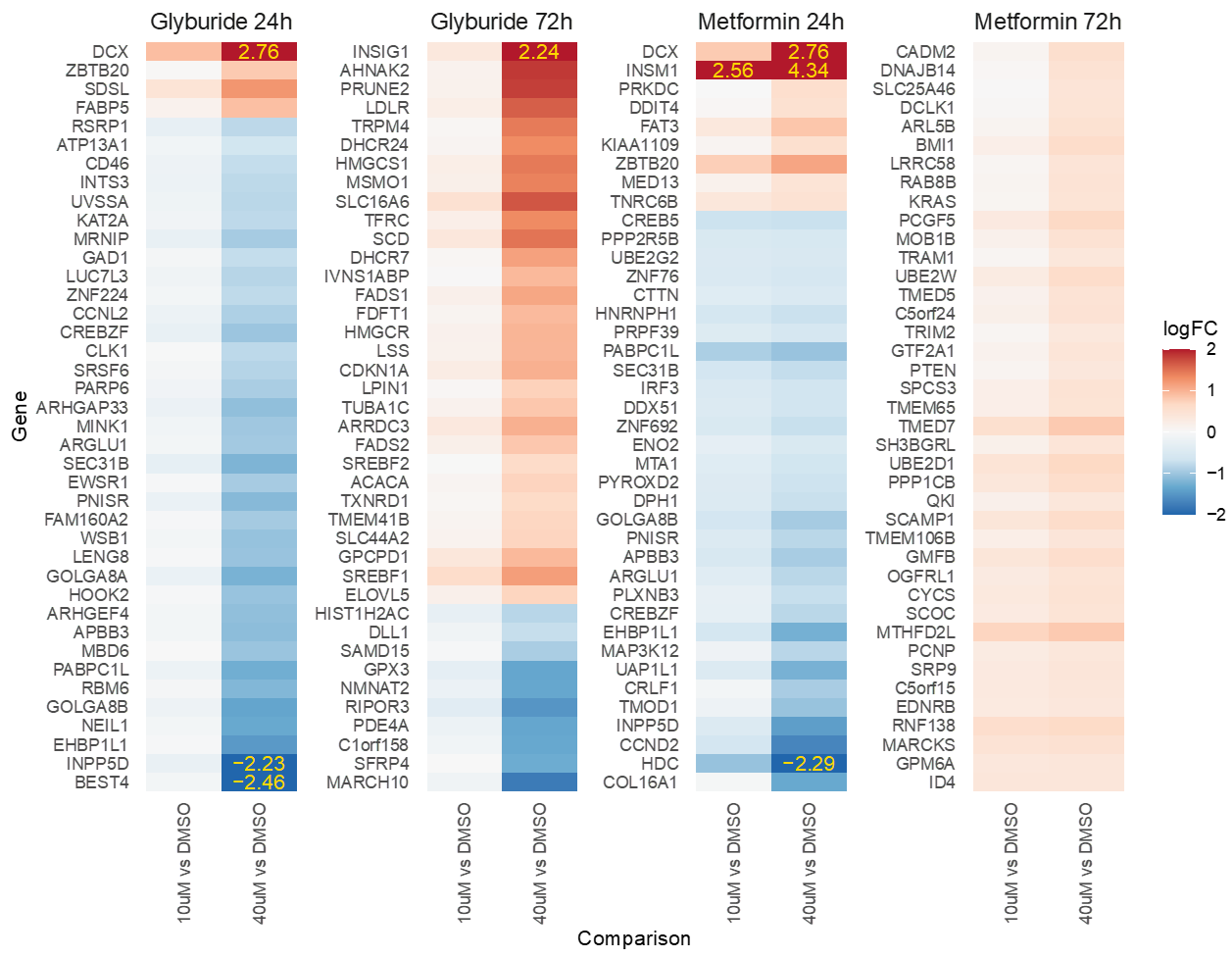


**Supplementary Figure 7 | Full list of genes with dose-dependent changes in expression for metformin and glyburide.** Drugs treated differentiated human neural ReNcells at two concentrations (10 or 40µM) for either 24h or 72h.


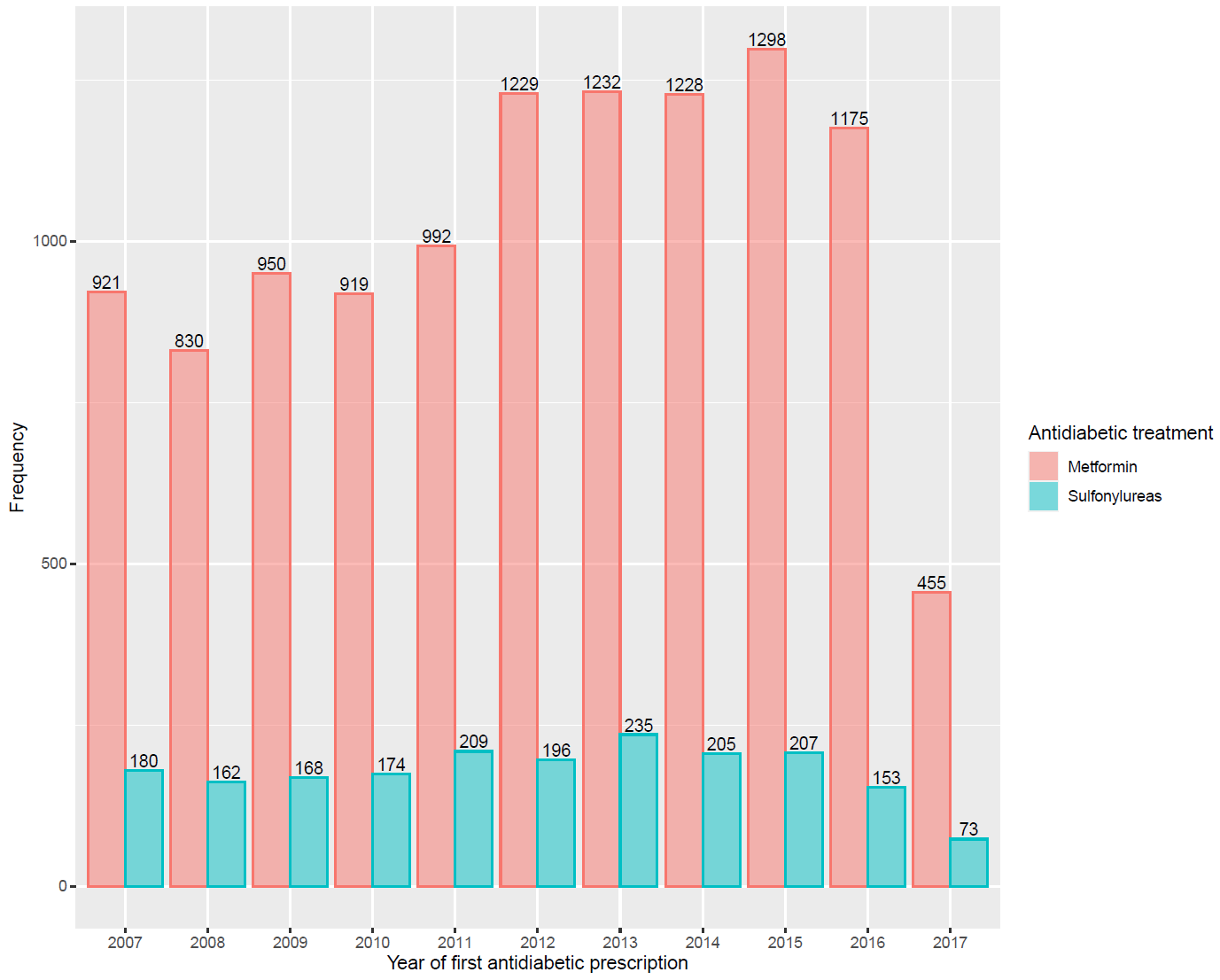


**Supplementary Figure 8 | Number of new prescriptions for metformin and sulfonylureas per**

**year in the US RPDR.**


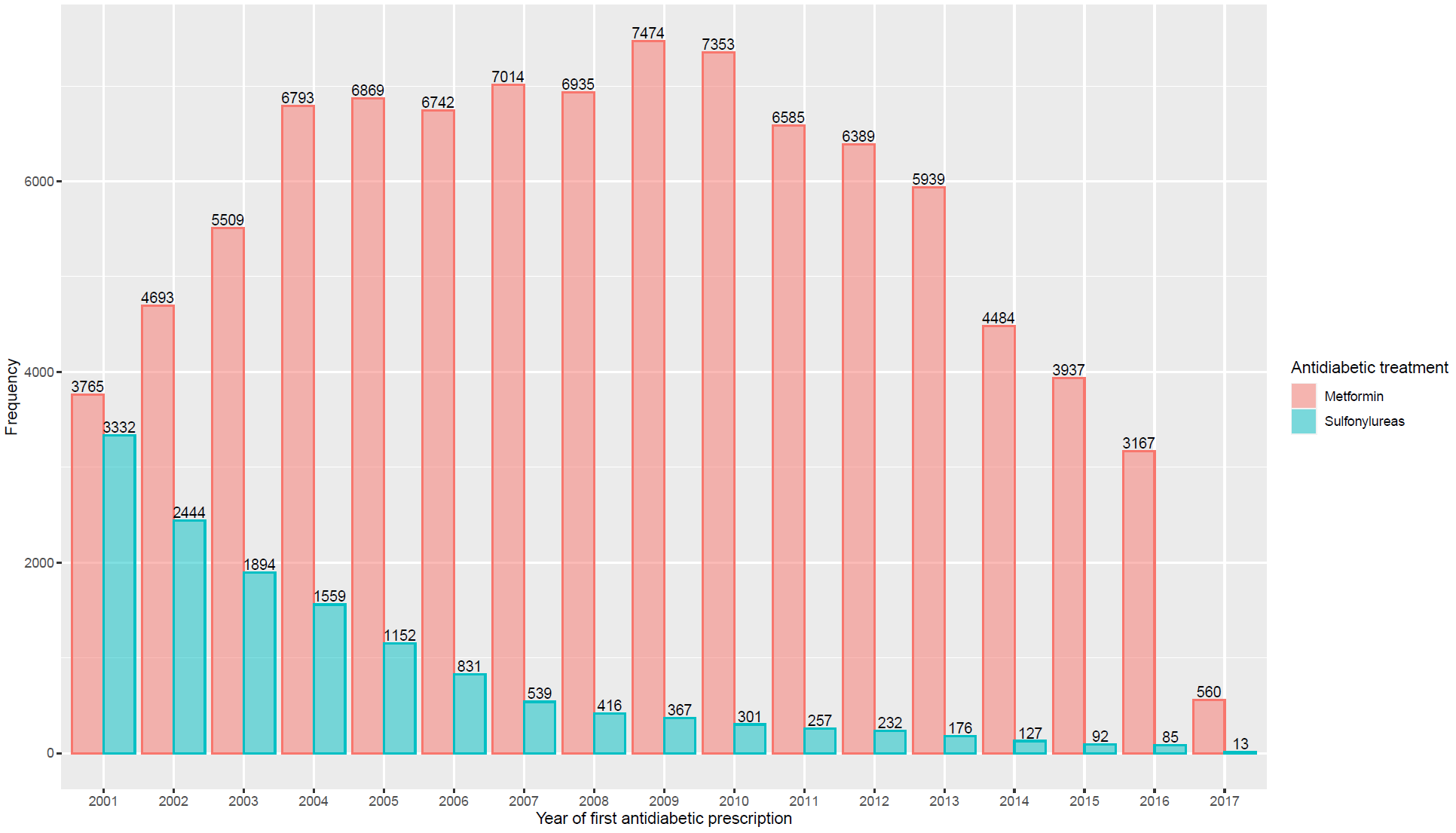


**Supplementary Figure 9 | Number of new prescriptions for metformin and sulfonylureas per year in the UK CPRD.**


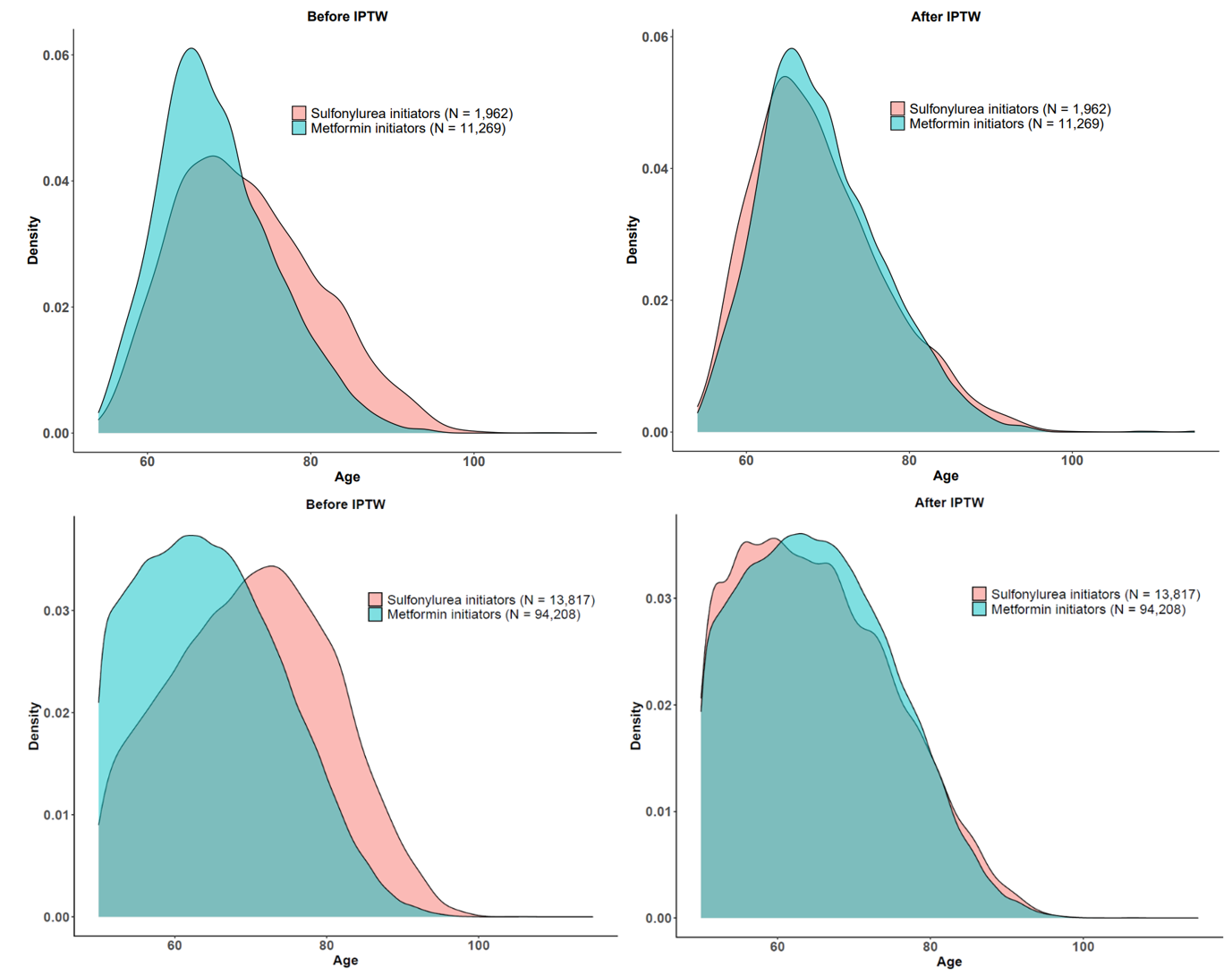


**Supplementary Figure 10 | Density curves for patient age at baseline, stratified by treatment group, before and after IPTW. (a)** US RPDR cohort. **(b)** UK CPRD cohort.

**
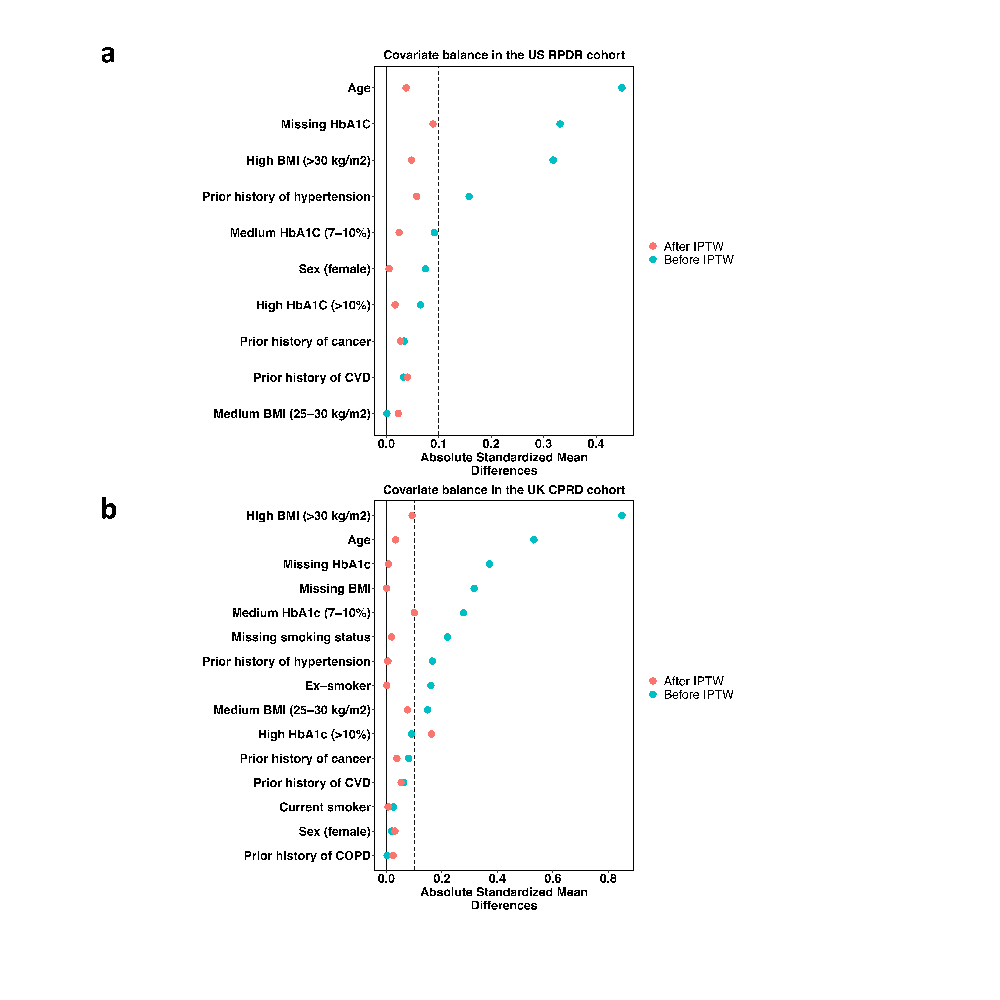
**

**Supplementary Figure 11 | Overall covariate balance before and after IPTW. (a)** US RPDR cohort. **(b)** UK CPRD cohort. The standardized mean difference^76^ is a measure of distance between two group means (for metformin and sulfonylurea initiators) in terms of one or more variables (definitions of standardized mean differences for both binary and continuous covariates were adopted from previous work^77^). It is often used in practice as a measure of balance of individual covariates before and after propensity score weighting. As it is standardized, comparison across variables on different scales is made possible.


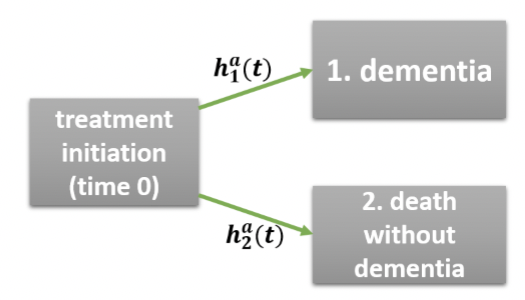


**Supplementary Figure 12 | A single-world competing risks model with two events, namely dementia and death prior to dementia.**

**EXTENDED DATA TABLES**

**Extended Data Table 1 | US CKD diagnosis codes / eGFR criterion table.**

Available on GitHub: <https://github.com/labsyspharm>.

**Extended Data Table 2 | UK CKD diagnosis codes.**

Available on GitHub: <https://github.com/labsyspharm>.

| Outcome event | Age categories (year) | US RPDR | | | |
| --- | --- | --- | --- | --- | --- |
|  |  | N | Cases | HR (95% CI) | P-value |
| All-cause mortality | ≤65 | 4669 | 187 | 0.58 (0.40-0.85) | 0.005 |
|  | >65 | 8522 | 562 | 0.58 (0.48-0.69) | <0.001 |
|  | ≤75 | 10614 | 478 | 0.53 (0.43-0.66) | <0.001 |
|  | >75 | 2577 | 271 | 0.63 (0.50-0.81) | <0.001 |
| Dementia incidence | ≤65 | 4669 | 273 | 0.57 (0.42-0.78) | <0.001 |
|  | >65 | 8522 | 837 | 0.96 (0.80-1.14) | 0.608 |
|  | ≤75 | 10614 | 677 | 0.77 (0.63-0.94) | 0.011 |
|  | >75 | 2577 | 433 | 0.90 (0.73-1.10) | 0.310 |

**Extended Data Table 3 | Subgroup analysis stratified by baseline age in US RPDR cohort.** A two-sided Wald test of whether the hazard ratio associated with metformin treatment initiation is 1, with robust variance estimator, was used. No further correction for multiple hypothesis testing was applied.

| Outcome event | Age categories (year) | UK CPRD | | | |
| --- | --- | --- | --- | --- | --- |
|  |  | N | Cases | HR (95% CI) | P-value |
| All-cause mortality | ≤65 | 52570 | 3851 | 0.53 (0.43-0.65) | <0.001 |
|  | >65 | 55455 | 14263 | 0.72 (0.67-0.77) | <0.001 |
|  | ≤75 | 89747 | 11008 | 0.60 (0.54-0.67) | <0.001 |
|  | >75 | 18278 | 7106 | 0.76 (0.71-0.82) | <0.001 |
| Dementia incidence | ≤65 | 52570 | 919 | 0.73 (0.51-1.05) | 0.091 |
|  | >65 | 55455 | 6341 | 0.89 (0.80-0.99) | 0.032 |
|  | ≤75 | 89747 | 3876 | 0.83 (0.70-0.98) | 0.026 |
|  | >75 | 18278 | 3384 | 0.88 (0.78-1.00) | 0.048 |

**Extended Data Table 4 | Subgroup analysis stratified by baseline age in the UK CPRD cohort.** Error bars represent 95% CIs for hazard ratios. A two-sided Wald test of whether the hazard ratio associated with metformin treatment initiation is 1, with robust variance estimator, was used. No further correction for multiple hypothesis testing was applied.

*Outcome: All-cause mortality*

| **Weighting scheme / Adjustment scheme** | **Cox PH without (age, sex)** | **Cox PH with (age, sex)** |
| --- | --- | --- |
| **ATE weights** | HR (ref. = sulf): 0.570  95% CI: (0.480; 0.677) | HR (ref. = sulf): 0.583 95% CI: (0.491; 0.693) |
| **ATT weights** | HR (ref. = sulf): 0.569 95% CI: (0.476; 0.680) | HR (ref. = sulf): 0.585 95% CI: (0.490; 0.699) |

*Outcome: Dementia onset*

| **Weighting scheme / Adjustment scheme** | **Cox PH without (age, sex)** | **Cox PH with (age, sex)** |
| --- | --- | --- |
| **ATE weights** | HR (ref. = sulf): 0.806  95% CI: (0.685; 0.949) | HR (ref. = sulf): 0.826 95% CI: (0.702; 0.971) |
| **ATT weights** | HR (ref. = sulf): 0.796 95% CI: (0.672; 0.944) | HR (ref. = sulf): 0.814 95% CI: (0.686; 0.967) |

*Outcome: Death (without dementia)*

| **Weighting scheme / Adjustment scheme** | **Cox PH without (age, sex)** | **Cox PH with (age, sex)** |
| --- | --- | --- |
| **ATE weights** | HR (ref. = sulf): 0.600  95% CI: (0.490; 0.735) | HR (ref. = sulf): 0.619 95% CI: (0.505; 0.757) |
| **ATT weights** | HR (ref. = sulf): 0.597 95% CI: (0.484; 0.736) | HR (ref. = sulf): 0.616 95% CI: (0.499; 0.760) |

**Extended Data Table 5 | Sensitivity analyses with respect to weighting scheme and further covariate adjustment in the structural outcome models for the US RPDR cohort.** “ref.=sulf” indicates that the reference group is patients initiating on sulfonylureas.

*Outcome: All-cause mortality*

| **Weighting scheme / Adjustment scheme** | **Cox PH without (age, sex)** | **Cox PH with (age, sex)** |
| --- | --- | --- |
| **ATE weights** | HR (ref. = sulf): 0.657  95% CI: (0.611; 0.707) | HR (ref. = sulf): 0.664 95% CI: (0.618; 0.714) |
| **ATT weights** | HR (ref. = sulf): 0.621 95% CI: (0.570; 0.676) | HR (ref. = sulf): 0.630 95% CI: (0.579; 0.686) |

*Outcome: Dementia onset*

| **Weighting scheme / Adjustment scheme** | **Cox PH without (age, sex)** | **Cox PH with (age, sex)** |
| --- | --- | --- |
| **ATE weights** | HR (ref. = sulf): 0.863  95% CI: (0.775; 0.961) | HR (ref. = sulf): 0.878 95% CI: (0.789; 0.977) |
| **ATT weights** | HR (ref. = sulf): 0.843 95% CI: (0.741; 0.958) | HR (ref. = sulf): 0.877 95% CI: (0.773; 0.995) |

*Outcome: Death (without dementia)*

| **Weighting scheme / Adjustment scheme** | **Cox PH without (age, sex)** | **Cox PH with (age, sex)** |
| --- | --- | --- |
| **ATE weights** | HR (ref. = sulf): 0.641  95% CI: (0.593; 0.694) | HR (ref. = sulf): 0.648 95% CI: (0.599; 0.700) |
| **ATT weights** | HR (ref. = sulf): 0.605 95% CI: (0.552; 0.662) | HR (ref. = sulf): 0.613 95% CI: (0.559; 0.672) |

**Extended Data Table 6 | Sensitivity analyses with respect to weighting scheme and further covariate adjustment in the structural outcome models for the UK CPRD cohort.** “ref.=sulf” indicates that the reference group is patients initiating on sulfonylureas.


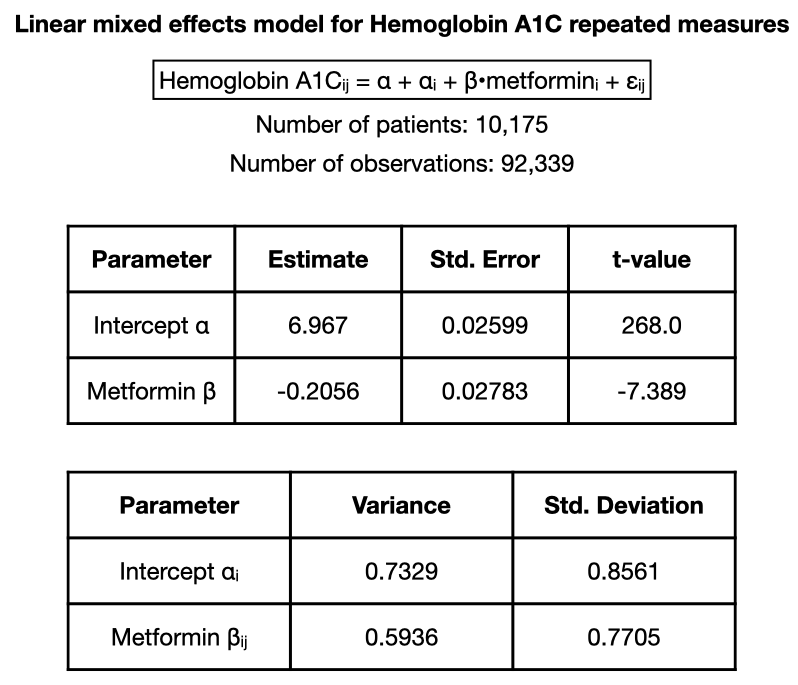


**Extended Data Table 7 | Tabulated results of repeated measures mixed effects model for HbA1C patient data post-baseline, in the US RPDR.** Estimates of fixed effects (above) and random effects (below) are provided. Difference in post-baseline HbA1C levels was not clinically significant (i = patient; j = observation).

**Extended Data Table 8 | Differential gene expression between drug and DMSO vehicle: metformin**

**24h and 72h; glyburide 24h and 72h and pathway analysis.**

Available on GitHub: <https://github.com/labsyspharm>.

| **Human Gene Name** | **logFC** |
| --- | --- |
| SPP1 (osteopontin) | -2.34745 |
| BRINP2 | -2.31312 |
| PTGDS (prostaglandin D2 synthetase) | -1.68581 |
| ADAM12 | -1.56585 |
| FAM20C (extracellular serine threonine kinase) | -1.22483 |
| TFRC | -1.16368 |
| CD44 | -1.13569 |
| TF | -1.07581 |
| ANGPTL2 | -0.99027 |
| NOG | 1.064732 |
| SFRP4 | 1.071367 |
| IL16 | 1.114638 |
| MATN2 | 1.166752 |
| GLB1L2 | 1.16997 |
| SLIT1 | 1.202582 |
| MASP1 | 1.239591 |
| COCH | 1.272685 |
| PI16 | 1.277536 |
| VSTM2L | 1.304698 |
| A2ML1 | 1.361365 |
| RELN | 1.409874 |
| CRISPLD2 | 1.464841 |
| TNR (Tenascin) | 1.574132 |
| LDLRAD2 (LDLR Associated D 2) | 1.820836 |
| CILP (Cartilage intermediate layer protein) | 2.070604 |
| VWA5B1 | 2.251385 |

**Extended Data Table 9 | Genes encoding secreted protein products whose RNA expression levels changed by more than 2x in response to exposure for 72 hours to 40 µM metformin relative to 40 µM glyburide in differentiated human ReNcells.** Gene names in gray were not detected in CSF proteomic analysis conducted by TMT-LC/MS (1) or Olink methodologies.

**Extended Data Table 10 | US dementia diagnosis codes.**

Available on GitHub: <https://github.com/labsyspharm>.

**Extended Data Table 11 | US dementia/antidiabetic drugs.**

Available on GitHub: <https://github.com/labsyspharm>.

**Extended Data Table 12 | UK dementia diagnosis codes.**

Available on GitHub: <https://github.com/labsyspharm>.

**Extended Data Table 13 | UK dementia/antidiabetic drug codes.**

Available on GitHub: <https://github.com/labsyspharm>.

**Methods**

|  | **Our study** | **Orkaby^30^** | **Scherrer^29^** |
| --- | --- | --- | --- |
| **Confounding by treatment indication** | Renal impairment addressed.  Stabilized IPTW. No trimming. | Renal impairment addressed.  Non-stabilized IPTW. Trimming at PS=20. | Renal impairment addressed.  Stabilized IPTW. Trimming at PS=10. |
| **Competing death** | Yes | No | No |
| **Sensitivity to outcome model structure (Cox PH)** | Yes, using a nonparametric approach | No | No |
| **Age-based stratification** | Dichotomous splits using 65, 70, and 75 as thresholds | Dichotomous split using 75 as a threshold | Categorical split:  50-64; 65-74; 75+ |
| **Handling of data missingness** | Patients with missing race/ethnicity were considered.  Missingness rates were as follow: UK CPRD (35%);  US RPDR (%).  Missing as a separate category for the following variables.  UK CPRD: IMD (7%), smoking status (2%), HbA1C (21%), and BMI (3%).  US RPDR: HbA1C (38%), BMI (32%). | Complete case analysis wrt to the following variables: race (used to calculate eGFR), HbA1C, BMI, and renal function (estimated via eGFR or presence of ICD codes). | Complete case analysis wrt to the following variables: creatinine and HbA1C, demographic characteristics. |
| **Outcome ascertainment** | **ICD-9/10 codes or dementia-related drugs**  ICD-9/10 codes:  290.X, 294.X, and 331.X; 780.93, G30.X, and G31.X  Dementia-related drugs: Donepezil, Galantamine,  Rivastigmine, and their respective brand names Aricept, Razadyne, Exelon | **ICD-9/10 codes only**  290.x, 291.2, 294.1, 294.11, 331.x (except 331.83  [MCI]), 333.0, 333.4, 797, 332.0, 294.8, 046.1, and 046.3 | **ICD-9/10 codes only**  290.0 290.1x 290.2x 290.3 290.4x 294.1x  294.2x 331.0 331.1x 331.2 331.82  331.83, used as a qualifying definition for dementia in sensitivity analysis |

**Results**

*Study population*

|  | **Our study** | **Orkaby** | **Scherrer** |
| --- | --- | --- | --- |
| **Age at treatment initiation** | Metformin: UK CPRD: US RPDR:  Sulfonylureas:  UK CPRD: US RPDR: | Older at treatment initiation (average: 73.5, +/- 5.9 years old) because an inclusion criterion imposed age $\geq$ 65 at new type 2 diabetes diagnosis | VHA: 60.8 years old KPW: 63.1 years old |
| **Sex** | UK CPRD (% male)  Metformin: 57.4%  Sulfonylureas: 58.3%  US RPDR (% male)  Metformin: 49.2%  Sulfonylureas: 52.9% | % male  Metformin: 98.8%  Sulfonylureas: 99.0% | % male  VHA: 96.8% KPW: 50.4% |
| **Calendar time** | UK CPRD: 2001-2017 US RPDR: 2007-2017 | 2001-2012 | VHA: 2002-2012 KPW: 1996-2012 |
| **Washout period** | One year | Two years | VHA: Two years KPW: No washout, continuous enrollment |
| **Follow-up** | UK CPRD median  Metformin: 7.0 years  Sulfonylureas: 7.0 years  US RPDR median:  Metformin: 5.3 years  Sulfonylureas: 5.3 years | Average of 5 years (+/-3.1) | VHA median  Metformin: 6.4 years  Sulfonylureas: 6.7 years  KPW median  Metformin: 6.1 years  Sulfonylureas: 7.3 years |

*Statistical analysis*

|  | **Our study** | **Orkaby** | **Scherrer** |
| --- | --- | --- | --- |
| **Overall HR** | UK CPRD:  HR = 0.86 (0.77-0.96)  US RPDR:  HR = 0.81 (0.69-0.94) | Not provided | VHA:  HR=0.93 (0.87–0.99)  KPW:  HR=0.89 (0.74–1.07)  Pooled analysis:  HR=0.92 (0.87–0.98) |
| $\leq$ **75** | UK CPRD: HR = 0.83 (0.70-0.98)  US RPDR: HR = 0.77 (0.63-0.94) | 0.89 (0.79–0.99)* | VHA*:  50-64 = 0.86 (0.76–0.98); 65-74 = 0.87 (0.80–0.96)  KPW*:  50-64 = 1.33 (0.78-2.25); 65-74 = 0.79 (0.58-1.07) |
| **>75** | UK CPRD:  HR = 0.88 (0.78-1.00)  US RPDR:  HR = 0.90 (0.73-1.10) | 0.96 (0.87–1.05)  *used <75 and $\geq$75 instead | VHA*: 75+ = 1.03 (0.9-1.14)  KPW*: 75+ = 0.79 (0.60-1.03) |

*In the study, the authors used <75 and $\geq$75, while we used $\leq$75 and >75.

**Extended Data Table 14 | Comparison of this study with related studies^29,30^.**
