# Supplementary material for: Causal inference in medical records and complementary systems pharmacology for metformin drug repurposing towards dementia
Source: Nat Commun. 2022 Dec 10;13:7652. doi: 10.1038/s41467-022-35157-w (PMC9741618; doi:10.1038/s41467-022-35157-w)
Supplement: Supplementary file 3 — Reporting Summary [file 41467_2022_35157_MOESM3_ESM.pdf]

nature portfolio

Corresponding author(s): Mark Albers, Deborah Blacker, Sudeshna Das, Ioanna Tzoulaki

Last updated by author(s): Nov 18, 2022

Reporting Summary

Nature Portfolio wishes to improve the reproducibility of the work that we publish. This form provides structure for consistency and transparency in reporting. For further information on Nature Portfolio policies, see our Editorial Policies and the Editorial Policy Checklist.

Statistics

For all statistical analyses, confirm that the following items are present in the figure legend, table legend, main text, or Methods section.

n/a	Confirmed	
☐	☒	The exact sample size (*n*) for each experimental group/condition, given as a discrete number and unit of measurement
☐	☒	A statement on whether measurements were taken from distinct samples or whether the same sample was measured repeatedly
☐	☒	The statistical test(s) used AND whether they are one- or two-sided<br>*Only common tests should be described solely by name; describe more complex techniques in the Methods section.*
☐	☒	A description of all covariates tested
☐	☒	A description of any assumptions or corrections, such as tests of normality and adjustment for multiple comparisons
☐	☒	A full description of the statistical parameters including central tendency (e.g. means) or other basic estimates (e.g. regression coefficient) AND variation (e.g. standard deviation) or associated estimates of uncertainty (e.g. confidence intervals)
☐	☒	For null hypothesis testing, the test statistic (e.g. *F*, *t*, *r*) with confidence intervals, effect sizes, degrees of freedom and *P* value noted<br>*Give P values as exact values whenever suitable.*
☒	☐	For Bayesian analysis, information on the choice of priors and Markov chain Monte Carlo settings
☐	☒	For hierarchical and complex designs, identification of the appropriate level for tests and full reporting of outcomes
☒	☐	Estimates of effect sizes (e.g. Cohen's *d*, Pearson's *r*), indicating how they were calculated

Our web collection on statistics for biologists contains articles on many of the points above.

Software and code

Policy information about availability of computer code

Data collection	The data used in the study are from two large-scale EHR databases: the Research Patient Data Registry in the United States at Mass GeneralBrigham Healthcare (US RPDR) and the Clinical Practice Research Datalink in the United Kingdom (UK CPRD).<br>US RPDR. The study cohort was selected from the RPDR registry13. RPDR is a longitudinal centralized clinical data registry with ~G.s million patients mainly from the Boston area as of June 2020. The data are collected from EHR systems within Mass General Brigham (MGB) Healthcare (formerly Partners Healthcare), comprising two major academic hospitals, as well as several community hospitals and community health centers in the Boston area. Death records are updated periodically from the US Social Security Death Index (2007-2017). Use of RPDR data for this study was approved by the institutional review board (IRB) of MGB.<br>UK CPRD. The study cohort was selected from the CPRD database. CPRD is a longitudinal national primary care database, with ~17 million patients from 13 regions across the UK. The data are collected from EHR systems in general practice physician (GP) practices. GP practices can enroll the CPRD data system on an ongoing basis and can leave it at any time. Over 700 GP practices {8% of total GP practices) have contributed data to CPRD and the mean follow-up time for patients included in CPRD is around eight years. Death records are updated periodically from the UK Office of National Statistics (ONS). Additionally, data linkages were established with secondary care data from Hospital Episode Statistics (HES) as well as with small-area measures of social deprivation (2001-2017). Use of CPRD data for this study was approved by the Independent Scientific Advisory Committee (ISAC) for Medicines and Healthcare products Regulatory Agency (MHRA) database research (protocol number: 19_065R).<br>Both the US RPDR and the UK CPRD data includes patient demographics; encounter details such as dates, providers, diagnoses, and procedures; medical notes, drug prescriptions, and laboratory test results. We reported our findings according to the RECORD reporting guidelines.
Data analysis	EHR data analysis: The R package, causalCmprsk, developed for the competing risks analysis is available at CRAN,v. 1.0.3<br>Differentiated human neural cell RNA-seq data analysis: Differential gene expression between compound-treated samples and DMSO controls

was performed by the R package edgeR version 3.26.5. Genes were subsequently sorted by the resulting log-fold change values and queried against canonical pathways in the Molecular Signatures Database71 using Gene Set Enrichment Analysis.
Postmortem RNA-seq analysis: The methodology being used to measure and harmonize RNA-seq values across the brain samples has been previously described75 and can be found at https://github.com/Sage-Bionetworks/amp-rnaseq

For manuscripts utilizing custom algorithms or software that are central to the research but not yet described in published literature, software must be made available to editors and reviewers. We strongly encourage code deposition in a community repository (e.g. GitHub). See the Nature Portfolio guidelines for submitting code & software for further information.

Data

Policy information about availability of data

All manuscripts must include a data availability statement. This statement should provide the following information, where applicable:

- Accession codes, unique identifiers, or web links for publicly available datasets
- A description of any restrictions on data availability
- For clinical datasets or third party data, please ensure that the statement adheres to our policy

US RPDR. Researchers can obtain an anonymized version of the study dataset from the authors upon request and completion of the MGB Health data use agreement for the use of RPDR data. This agreement ensures the privacy of MGB patients and compliance with US regulatory standards and has been approved by the MGB IRB.
UK CPRD. According to the UK Data Protection Act, information governance restrictions (to protect patient confidentiality) prevent data sharing via public deposition. Therefore, CPRD data that support the findings of this study are not publicly available. Data extracts can be requested by applying to the Clinical Practice Research Datalink for data spanning the years 2000 to 2018 (https://www.cprd.com). The code to process the data is available from the authors upon request. All requests will be answered in 30 days or less.

Human research participants

Policy information about studies involving human research participants and Sex and Gender in Research.

Reporting on sex and gender	We used the term sex (biological attribute) throughout the manuscript. Sex was determined based on electronic health records. Our main analysis included both male (N=6560 in US RPDR and 62080 in UK CPRD) and female patients (N=6631 in US RPDR and 45945 in UK CPRD), and we also conducted a subgroup analysis by sex (Fig. 2b and 2d).
Population characteristics	Population characteristics are presented in Table 2 and described in the first section of Results
Recruitment	This study was reviewed by an Institutional Review Board. The IRB waived informed consent for this retrospective study. The CPRD study participants is retrospective research, and the patient records are anonymized. No recruitment is possible.
Ethics oversight	Use of RPDR data for this study was approved by the institutional review board (IRB) of MGB. Use of CPRD data for this study was approved by the Independent Scientific Advisory Committee (ISAC) for Medicines and Healthcare products Regulatory Agency (MHRA) database research (protocol number: 19_065R).

Note that full information on the approval of the study protocol must also be provided in the manuscript.

Field-specific reporting

Please select the one below that is the best fit for your research. If you are not sure, read the appropriate sections before making your selection.

[x] Life sciences   [ ] Behavioural & social sciences   [ ] Ecological, evolutionary & environmental sciences

For a reference copy of the document with all sections, see nature.com/documents/nr-reporting-summary-flat.pdf

Life sciences study design

All studies must disclose on these points even when the disclosure is negative.

Sample size	Sample size was determined based on previous experiments testing drugs in cell culture (75). Aall sample were done in triplicate.
Data exclusions	None
Replication	There were 3 technical replicates in the cell culture. All replicates were successful.
Randomization	The cell were arrayed in 96 well dishes. The drug were distributed using a drug dispenser that randomizes the drug delivery in the wells.
Blinding	Blinding was not relevant to our study since all readouts were objective (RNA-seq) or ELISA and did not require any subjective interpretation, such as phenotypes under the microscope or manual cell counting.

Reporting for specific materials, systems and methods

We require information from authors about some types of materials, experimental systems and methods used in many studies. Here, indicate whether each material, system or method listed is relevant to your study. If you are not sure if a list item applies to your research, read the appropriate section before selecting a response.

Materials & experimental systems

n/a	Involved in the study
☒	☐ Antibodies
☐	☒ Eukaryotic cell lines
☒	☐ Palaeontology and archaeology
☒	☐ Animals and other organisms
☒	☐ Clinical data
☒	☐ Dual use research of concern

Methods

n/a	Involved in the study
☒	☐ ChIP-seq
☒	☐ Flow cytometry
☒	☐ MRI-based neuroimaging

Eukaryotic cell lines

Policy information about cell lines and Sex and Gender in Research

Cell line source(s)	ReN VM Cells - purchased from Thermo Fisher
Authentication	Cell line was not authenticated in this study. Full proteomics of cell line was published in 2019 (PMID 30778261)
Mycoplasma contamination	All cells tested negative for mycoplasma.
Commonly misidentified lines (See ICLAC register)	No commonly misidentified cell lines were used in the study
